# Alternaria alternata botybirnavirus 1 (AaBRV1) Infection Affects the Biological Characteristics of Its Host Fungus *Alternaria alternata*

**DOI:** 10.3390/jof11050376

**Published:** 2025-05-15

**Authors:** Xinyi Zhang, Qiqi Zhu, Ziyuan Chen, Ju Chen, Zhijun Liu, Xuehong Wu

**Affiliations:** 1 Sanya Institute of China Agricultural University, Yazhou District, Sanya 572025, China; zxy18511290531@163.com (X.Z.); zhijunl0103@126.com (Z.L.); 2 College of Plant Protection, China Agricultural University, Haidian District, Beijing 100193, China; 15817939173@163.com (Q.Z.); spadelychen@163.com (Z.C.); 18737152291@163.com (J.C.)

**Keywords:** Alternaria alternata botybirnavirus 1, biological characteristics, transcriptome, fungicide sensitivity, MFS transporters, Cytochrome P450

## Abstract

A botybirnavirus, Alternaria alternata botybirnavirus 1 (AaBRV1), had been identified from *Alternaria alternata* strain SD-BZF-19 isolated from diseased watermelon leaves in our previous study. In the current study, AaBRV1 was eliminated from its host fungus strain SD-BZF-19 using single hyphal tip culture method combined with high-temperature treatment to obtain the AaBRV1-free strain, which was named SD-BZF-19-G14. Compared with strain SD-BZF-19-G14, following AaBRV1 infection, colony color of strain SD-BZF-19 changed, and colony growth rate, dry weight of mycelial biomass, and sensitivity to difenoconazole, fludioxonil, and tebuconazole of strain SD-BZF-19 all decreased. However, the virulence of strain SD-BZF-19 was not significantly different from strain SD-BZF-19-G14, with disease index of watermelon leaves inoculated with SD-BZF-19 and SD-BZF-19-G14 being 90.83 and 81.67, respectively. A total of 1244 differentially expressed genes (DEGs) were identified in a comparative transcriptome analysis between the two strains, SD-BZF-19 and SD-BZF-19-G14. Relative to strain SD-BZF-19, the number of upregulated and downregulated DEGs in strain SD-BZF-19-G14 was 660 and 584, respectively. Notably, Pfam database annotated that the number of DEGs related to Major Facilitator Superfamily (MFS) and Cytochrome P450 (CYP450) was 36 and 28, respectively. To our knowledge, this is the first documentation of biological characteristics induced by AaBRV1 infection in *A. alternata*.

## 1. Introduction

“Botybirnaviridae” was originally proposed in 2013. The ICTV approved the creation of a new genus *Botybirnavirus* within this proposed family “Botybirnaviridae” in 2016. Finally, the family *Botybirnaviridae* was officially established in 2023 [1]. A total of 14 botybirnaviruses have been reported, and their detailed information was listed in Table S1 [2,3,4,5,6,7,8,9,10,11,12,13,14,15,16,17].

Among the 14 botybirnaviruses, nine of them have been recorded to affect biological characteristics of their hosts. BpRV1 could reduce radial growth rate and virulence of its fungal hosts and could cause abnormal colony morphology and membranous vacuoles/vesicles of its host Bc-72 [2]. SsBRV1 could transfect the protoplasts of the virus-free strain Ep-1PNA367; sometimes, the dsRNA3 (satellite-like RNA, SatlRNA) segment was eliminated in some transfectants. There was no significant difference in the colony morphology between the virus-free strain Ep-1PNA367 and these transfectants regardless of whether dsRNA3 was present in them or not. However, SsBRV1 could significantly reduce the growth rate and virulence of the transfectants with the dsRNA3 being present in them. No significant difference was found in growth rate and virulence between the virus-free strain Ep-1PNA367 and the transfectants without SatlRNA [3]. SsBRV2 could be introduced into virus-free *S. sclerotiorum* strain Ep-1PNA367R, and the transfectant was named Ep-1PNA367RVT. Compared with virus-free strain Ep-1PNA367R, Ep-1PNA367RVT showed obvious hypovirulent phenotypic traits, including lower growth rate, less virulence, and no sclerotia production [4]. BmBRV1-BdEW220 reduced the growth of *B. dothidea* and conferred hypovirulence to the fungal host [8]. *L. biglobosa* strain GZJS19 infected by LbBV1 did not show any significant phenotypical changes, compared with the LbBV1-free strains (including GZJS19-VF and four other field strains of *L. biglobosa*). However, different from the asymptomatic nature of LbBV1 infection in *L. biglobosa*, LbBV1 infection in *B. cinerea* strain t-459 resulted in its inability to produce sclerotia on PDA and conferred hypovirulence [12]. When AaBbV1 was introduced into a virus-free Japanese Ally-12 strain of *A. alternata* by protoplast fusion technique, the strain was asymptomatic [9]. Similarly, SsBRV3/SZ-150 could not induce phenotypic changes in its host [11]. Both DtBRV1-infected parent strain CJP4-1 and DtBRV1-transfected strains showed a latent infection, which revealed the infection of DtBRV1 had no phenotypic changes on its host, including colony morphology, growth rate, and virulence [14]. Compared with the AaBRV1-AT1-free strain TJ-NH-51S-4-VF, colony growth rate, spore production, and virulence of the *A. tenuissima* strain TJ-NH-51S-4 infected by AaBRV1-AT1 increased, but its sensitivity to difenoconazole decreased. Additionally, the expression level of one DEG-encoded cytochrome P450 (CYP450) sterol 14α-demethylase and four DEGs-encoded siderophore iron transporters were significantly upregulated in strain TJ-NH-51S-4 [17]. Collectively, the effects of botybirnaviruses on their hosts might be hypovirulent, hypervirulent, or latent.

Difenoconazole (demethylase inhibitor fungicide, DMI) [18], fludioxonil (quinone outside inhibitor fungicide, QoI) [19], and tebuconazole (DMI) [20] had been documented to effectively inhibit mycelial growth of *Alternaria*. Mycoviruses could increase/decrease the sensitivity of their hosts to fungicides. Alternaria alternata chrysovirus 1 (AaCV1-AT1) infection decreased the sensitivity of *A. tenuissima* strain SD-BZF-12 to difenoconazole and tebuconazole [21]. AaBRV1-AT1 infection reduced the sensitivity of *A. tenuissima* strain TJ-NH-51S-4 to difenoconazole [17]. Fusarium oxysporum virus 1-FON (FoV1-FON) infection significantly decreased the sensitivity of *Fusarium oxysporum* f. sp. *niveum* strain X-GS16 to difenoconazole, prochloraz, and pydiflumetofen [22]. Fusarium oxysporum alternavirus 1-FOM (FoAV1-FOM) infection increased the sensitivity of *F. oxysporum* f. sp. *melonis* strain T-BJ17 to difenoconazole and pydiflumetofen [23]. Pythium ultimum RNA virus 2 (PuRV2) infection increased the sensitivity of *G. ultimum* to metalaxyl [24]. Diaporthe pseudophoenicicola chrysovirus 1 (DpCV1) infection increased the sensitivity of *D. pseudophoenicicola* strain WC02 to prochloraz [25]. At present, there was no reports related with the effect of mycoviruses on the sensitivity of fungal host to fludioxonil.

The change of fungicide sensitivity may be related to the absence or presence of a sensitive target site; besides this, the overexpression of drug transporters located in the membranes of fungi can reduce the sensitivity of fungus to fungicides [26]. Major Facilitator Superfamily (MFS) is an important subfamily related to drug transport, which comprises a large group of secondary active transporters, mainly involved in a variety of physiological processes including nutrient uptake, metabolite efflux, and drug resistance [27]. CYP450 plays a role in metabolic processes in fungi, including sterol biosynthesis and the detoxification of exogenous compounds. These processes are closely related to fungicide resistance, particularly ergosterol, which is an essential component of fungal cell membranes [28].

Alternaria alternata botybirnavirus 1 (AaBRV1) was isolated from the *A. alternata* strain SD-BZF-19, the causal agent of watermelon leaf blight, in our previous research [16,29]. However, the impact of AaBRV1 on the biological characteristics of its host fungus *A. alternata* strain SD-BZF-19 has not been determined. In this study, the effect of AaBRV1 infection on colony color, colony growth rate, mycelial biomass, virulence, and sensitivity to difenoconazole, fludioxonil, and tebuconazole of its host fungus *A. alternata* strain SD-BZF-19 were evaluated. Additionally, a comparative analysis of transcriptome data was utilized to identify DEGs between AaBRV1-infected strain SD-BZF-19 and AaBRV1-free strain SD-BZF-19-14.

## 2. Materials and Methods

### 2.1. Fungal Strains and Culture Conditions

The AaBRV1-infected *A. alternata* strain SD-BZF-19 was isolated from diseased watermelon leaves with the symptom of leaf spot [16,29]. SD-BZF-19-G14 was an AaBRV1-free strain obtained by eliminating the mycovirus AaBRV1 from strain SD-BZF-19. The two strains (SD-BZF-19 and SD-BZF-19-G14) of *A. alternata* were grown on potato dextrose agar (PDA) plates in the dark at 25 °C for 7 d prior to their use.

### 2.2. Elimination of AaBRV1 from Strain SD-BZF-19

Single hyphal tip culture method combined with high-temperature treatment was used to eliminate AaBRV1 from strain SD-BZF-19 [23,30]. The strain SD-BZF-19 was incubated on PDA plates at 25 °C in the dark for 3 d and then transferred to 35 °C in the dark. After incubating for 10 d, single hyphal tips were transferred on new PDA plates and sub-cultured continuously until the virus-free strain was obtained. RT-PCR was employed to ascertain the efficacy of the elimination of AaBRV1 from SD-BZF-19 at every third subculture. The virus-free strain was sub-cultured on PDA medium for three times at 25 °C in the dark, and these cultures were detected by RT-PCR to ensure that the mycovirus was completely eliminated.

The collected mycelia of the subcultures derived from SD-BZF-19 were subjected to the extraction of total RNA using TRIpure Reagent (Aidlab Biotechnology, Beijing, China) following the manufacture instructions, which was used as a template to conduct RT-PCR using AaBRV1-specific primers RT-2-1-F (5′-AAACGAGGTATGTGAGGT-3′) and RT-2-1-R (5′-TTGCTTTCTTACTGGGTG-3′) designed based on complete nucleotide sequence of AaBRV1 (GenBank accession numbers: MK256972 and MK256973) [16] using Primer Premier Version 5.0 (PREMIER Biosoft International, Palo Alto, CA, USA) [31]. Gel electrophoretic profiles of RT-PCR products were performed to confirm whether AaBRV1 was eliminated from strain SD-BZF-19 successfully.

### 2.3. Observation of Colony Color and Measurements of Colony Growth Rate, Mycelial Biomass, and EC_50_ Value

In the present study, the sporulation ability or spore morphology were not assessed, which was due to that the two isogenic strains, SD-BZF-19 and SD-BZF-19-G14, could not produce spores. To assess the effect of the mycovirus AaBRV1 on biological characteristics (colony color, colony growth rate, and mycelial biomass) of the fungal host, the two strains, SD-BZF-19 and SD-BZF-19-G14, were individually cultured on PDA for 5 d at 25 °C in the dark. Mycelial agar plugs (5 mm in diameter) punched from the colony margin of each strain were placed on fresh PDA plates and incubated at 25 °C in the dark. The observation of colony color was carried out at 7 d after incubation, as previously described [21]. The colony diameters were measured at 8 d after incubation with three biological replicates. The dry weight of mycelial biomass experiment was conducted in accordance with previously described methods with three biological replicates [32]. Briefly, the mycelial plugs (5 mm in diameter) were placed in 100 mL potato dextrose broth (PDB). The fungal hyphae were filtered, collected, and washed after culturing for 7 d at 25 °C, which were placed at 55 °C for 24 h until the weight remained unchanged, which was regarded as the dry weight of mycelial biomass.

The sensitivity of the two strains, SD-BZF-19 and SD-BZF-19-G14, to difenoconazole, fludioxonil, and tebuconazole were evaluated in vitro as described in a previous study with minor modifications [17]. The PDA media were amended with three fungicides to establish final concentrations of 5.00, 1.00, 0.50, 0.10, and 0.05 µg/mL. PDA plates without fungicides were used as a control. The mycelial plugs (5 mm in diameter) from the colony margin of each strain were incubated in PDA plates with different concentrations of the three fungicides at 25 °C in the dark for 7 d, then the colony diameter was measured at two perpendicular axes. The median effective concentration (EC_50_) of these three fungicides for the two strains was calculated using probit analysis and linear regression of growth inhibition against the logarithmic values of concentrations of these three fungicides [21]. Three replicates were used for each strain-fungicide combination, and the experiment was repeated three times.

### 2.4. Pathogenicity Assay

Pathogenicity test of the two strains, SD-BZF-19 and SD-BZF-19-G14, was conducted on detached, fully expanded healthy watermelon (cv. Huaxin) leaves following the methodology described by Ma et al. [21]. Briefly, the two strains were grown on PDA plates at 25 °C in the dark for 7 d, and then agar plugs (5 mm in diameter) were cut from the edge of a colony and placed directly on the stabbed parts of the upper surface of these fully expanded watermelon leaves (Two agar plugs were inoculated on each leaf). Ten inoculated watermelon leaves were then placed in a growth chamber at 25 °C, 90% relative humidity (RH), and a 12 h photoperiod per day. The diameter of lesions on watermelon leaves was measured at 7 d post-inoculation, and these values were utilized to calculate the disease incidence and disease index for the two strains. Disease severity (DS) was scored on a 5-point rating system according to Ma et al. [29]: 0 = no lesion, 1 = lesions < 1 mm in diameter, 2 = lesions 1 to 5 mm in diameter, 3 = lesions 5 to 10 mm in diameter, and 4 = lesions >10 mm in diameter. The experiment was repeated three times.

### 2.5. cDNA Library Preparation and Transcriptomic Analysis

SD-BZF-19 and SD-BZF-19-G14 were cultured on the PDA plates for 7 d with three replicates. For each replication, 0.2 g of mycelia was collected for RNA extraction. The RNA samples were sent to Biomarker Technologies Co., Ltd. (Beijing, China) for transcriptome sequencing. RNA concentration and purity were measured using NanoDrop 2000 (Thermo Fisher Scientific, Wilmington, DE, USA). RNA integrity was assessed using the RNA Nano 6000 Assay Kit of the Agilent Bioanalyzer 2100 system (Agilent Technologies, Santa Clara, CA, USA). After testing quality, a total amount of 1 μg RNA per sample was used as input material for the RNA sample preparations. Sequencing libraries were generated using Hieff NGS Ultima Dual-mode mRNA Library Prep Kit for Illumina (Yeasen Biotechnology (Shanghai) Co., Ltd., Shanghai, China) and the libraries were sequenced on an Illumina NovaSeq platform to generate 150 bp paired-end reads. Clean data (clean reads) were obtained by removing reads containing adapter, reads containing ploy-N, and low-quality reads from raw data. Clean reads were mapped to the reference genome of *A. alternata* SRC1lrK2f (GCA_001642055) (https://www.ncbi.nlm.nih.gov/datasets/genome/GCF_001642055.1/, accessed on 30 January 2025) by the software of Hisat2 version 2.0.4 [33]. Gene function was annotated based on the following databases: Nr (NCBI non-redundant protein sequences) [34], Pfam (Protein family) [35], KOG [36]/COG [37] (Clusters of Orthologous Groups of proteins), Swiss-Prot (A manually annotated and reviewed protein sequence database) [38], KO (KEGG Ortholog database) [39], and GO (Gene Ontology) [40]. DEGs from SD-BZF-19 and SD-BZF-19-G14 were performed using the DESeq2 version 1.30.1 [41]. The *p*-value < 0.01 & Fold Change ≥ 2 was set as the threshold for significantly differential expression. The related data were deposited in the NCBI Sequence Read Archive (SRA) database under the accession number PRJNA1250114.

### 2.6. Validation of Transcriptome Data Using Reverse Transcription-Quantitative PCR

Reverse transcription-quantitative PCR (RT-qPCR) was performed to validate the expression of 10 randomly selected DEGs (Table S2) using gene-specific primers (Table S3), which were designed based on the transcriptome data utilizing Primer Premier Version 5.0. Total RNA extracted from the two strains (SD-BZF-19 and SD-BZF-19-G14) were used as templates to synthesize first strand of cDNA using PrimeScript™ RT reagent Kit with gDNA Eraser (Perfect Real Time) (Code No. RR047A) of TaKaRa Bio Inc. (Kusatsu, Japan) according to the manufacture instructions. RT-qPCR was conducted using a TB Green^®^ *Premix Ex Taq*™ (Tli RNaseH Plus) (Code No. RR420A) of TaKaRa Bio Inc. (Kusatsu, Japan) according to the manufacturer’s protocols. The histone 3 gene (*HIS3*) was used as an internal control [17]. The primer specificity was tested by ordinary RT-PCR and observed by melt curve plot. The cycle threshold (CT) values controlled ranged from 15 to 35 by diluting the cDNA to ensure that the data was available. Fold changes observed ranged from 0.10 to 14.99 (Average fold changes were listed in Table S2). The RT-qPCR analysis utilized three biological replicates and was conducted three times. The relative expression of each gene was calculated using 2^−∆∆CT^ method [42].

### 2.7. Statistical Analysis

The statistical significance of colony growth rate, dry weight of mycelial biomass, EC_50_ values of difenoconazole, fludioxonil, and tebuconazole, disease incidence and disease index between SD-BZF-19 and SD-BZF-G14 were analyzed with GraphPad Prism version 9.0 software (GraphPad Software, Inc., Boston, MA, USA). The assumptions of normality were evaluated by Shapiro–Wilk test, and unpaired *t*-test was used to compare the data between the two groups (ns, no significant; *, *p* < 0.05; **, *p* < 0.01; ***, *p* < 0.001; ****, *p* < 0.0001).

## 3. Results

### 3.1. Effect of AaBRV1 on Colony Color, Growth Rate, and Mycelial Biomass of Its Host Fungus

**Figure 1:**
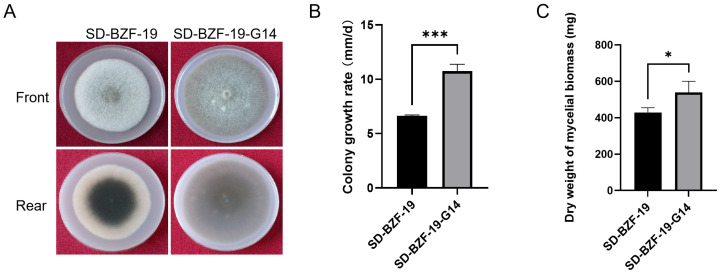
The effect of AaBRV1 on colony color, growth rate, and dry weight of mycelial biomass of its host fugus *A. alternata* strain SD-BZF-19. (**A**) Colony color of the two strains, SD-BZF-19 and SD-BZF-19-G14, cultured on PDA plates at 25 °C for 7 d in the dark. (**B**) Colony growth rate of the two strains, SD-BZF-19 and SD-BZF-19-G14, at 8 d after incubation. (**C**) Average dry weight of the two strains, SD-BZF-19 and SD-BZF-19-G14, cultured in potato dextrose broth (PDB) for 7 d at 25 °C in the dark. Stars indicate different levels of significant difference between the two strains as determined by the *t*-test using GraphPad Prism version 9.0 software (*, *p* < 0.05; ***, *p* < 0.001).

At the end of the 12th subculture, the AaBRV1-free strain SD-BZF-19-G14 was obtained using the single hyphal tip culture method combined with high temperature treatment to successfully eliminate AaBRV1 from *A. alternata* strain SD-BZF-19. The front colonies of the two strains, SD-BZF-19 and SD-BZF-19-G14, were grayish white and dark brown, respectively. The central colonies of strain SD-BZF-19 was dark green, and the edge of colonies of strain SD-BZF-19 was white when it was observed from the back view, while the central and edge of colonies of strain SD-BZF-19-G14 were both brown when it was observed from the back view (Figure 1A).

The average colony growth rate of strain SD-BZF-19 (7.76 mm/d) was significantly lower than that of strain SD-BZF-19-G14 (10.50 mm/d) (Figure 1B). Average dry weight of mycelial biomass generated by strain SD-BZF-19 (428.8 mg) was significantly lower than that of the mycelial biomass generated by strain SD-BZF-19-G14 (539.2 mg) (Figure 1C).

The collective effect of AaBRV1 infection on the phenotype of its host fungus *A. alternata* strain SD-BZF-19 includes changed colony color, reduced colony growth, and decreased mycelial biomass (Figure 1A–C).

### 3.2. Effect of AaBRV1 on Virulence of Its Host Fungus

**Figure 2:**
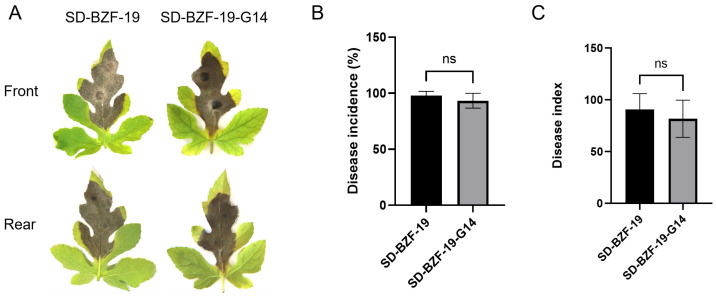
The effect of AaBRV1 on virulence of its host fungus *A. alternata* strain SD-BZF-19. (**A**) Disease symptoms on watermelon leaves inoculated with the two strains, SD-BZF-19 and SD-BZF-19-G14. (**B**) Disease incidence on watermelon leaves inoculated with the two strains, SD-BZF-19 and SD-BZF-19-G14, at 7 d post inoculation. (**C**) Disease index on watermelon leaves inoculated with the two strains, SD-BZF-19 and SD-BZF-19-G14, at 7 d post-inoculation. Stars indicate different levels of significant difference between the two strains as determined by the *t*-test using GraphPad Prism version 9.0 software (ns, no significant).

Watermelon leaves inoculated with the two strains, SD-BZF-19 and SD-BZF-19-G14, all exhibited dark brown lesions at 7 d post-inoculation (Figure 2A). The disease incidence and the disease index of watermelon leaves inoculated with strain SD-BZF-19 (97.78% and 90.83, respectively) was slightly higher than those of watermelon leaves inoculated with strain SD-BZF-19-G14 (93.33% and 81.67, respectively) (Figure 2B,C), but there was no statistical difference in the disease incidence and the disease index between these two strains.

### 3.3. Effect of AaBRV1 on Sensitivity to Three Fungicides of Its Host Fungus

**Figure 3:**
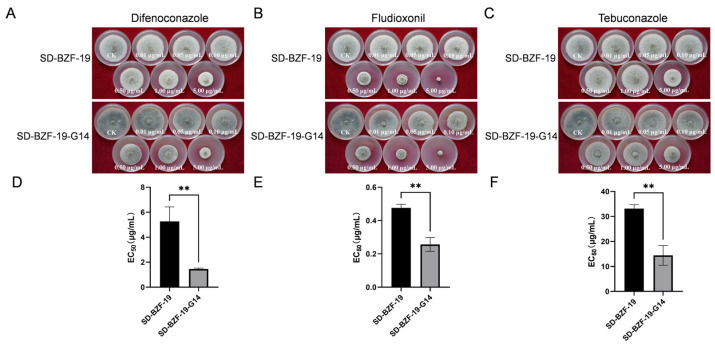
Sensitivity of two strains of *A. alternata*, SD-BZF-19 and SD-BZF-19-G14, to difenoconazole, fludioxonil, and tebuconazole. (**A**) Effect of difenoconazole on colony growth of the two strains, SD-BZF-19 and SD-BZF-19-G14. (**B**) Effect of fludioxonil on colony growth of strains SD-BZF-19 and SD-BZF-19-G14. (**C**) Effect of tebuconazole on colony growth of the two strains, SD-BZF-19 and SD-BZF-19-G14. (**D**) Median effective concentration (EC_50_) of difenoconazole against the two strains, SD-BZF-19 and SD-BZF-19-G14. (**E**) EC_50_ of fludioxonil against the two strains, SD-BZF-19 and SD-BZF-19-G14. (**F**) EC_50_ of tebuconazole against the two strains, SD-BZF-19 and SD-BZF-19-G14. Stars indicate different levels of significant difference between the two strains as determined by the *t*-test using GraphPad Prism version 9.0 software (**, *p* < 0.01).

Three fungicides (difenoconazole, fludioxonil, and tebuconazole) all could inhibit mycelial growth of both SD-BZF-19 and SD-BZF-19-G14 (Figure 3A–C); however, the EC_50_ value of difenoconazole, fludioxonil, and tebuconazole against strain SD-BZF-19 (5.2690 µg/mL, 0.4764 µg/mL, and 33.4453 µg/mL, respectively) were significantly higher than that of difenoconazole, fludioxonil, and tebuconazole against SD-BZF-19-G14 (1.4589 µg/mL, 0.2568 µg/mL, and 14.4062 µg/mL, respectively) (Figure 3D–F). The results indicated that the sensitivity of strain SD-BZF-19 to these three fungicides dramatically decreased following AaBRV1 infection.

### 3.4. Overview of Differentially Expressed Genes (DEGs)

Six samples, including three samples of SD-BZF-19 (three biological replicates, namely OS-1, OS-2, and OS-3) and three samples of SD-BZF-19-G14 (three biological replicates, namely VF-1, VF-2, and VF-3) were sequenced to explore the effect of AaBRV1 on gene expression in its host fungus.

A total of 37.91 Gb clean data was obtained, and the percentage of Q30 bases in each sample was not less than 96.79%. Among them, 36,183,361~40,344,549 clean reads were compared to the reference genome of *A. alternata*, accounting for 88.56%~90.39% (Table 1). These results indicated that the sequencing data were reliable for further analysis.

**Figure 4:**
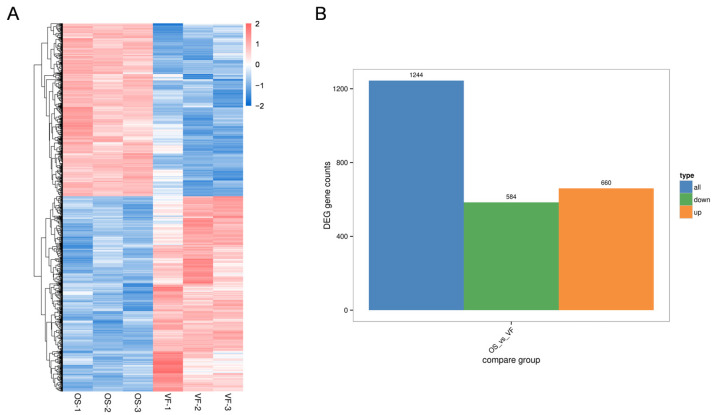
Hierarchical clustering and number of DEGs in the two strains, SD-BZF-19 and SD-BZF-19-G14. (**A**) Hierarchical clustering of DEGs in the two strains, SD-BZF-19 and SD-BZF-19-G14. Red and blue colors indicate varying degrees of up-regulated and down-regulated genes, respectively. Expression key is indicated on the upper right side of the figure. (**B**) A total of 1244 DEGs were identified, of which 660 DEGs were upregulated, and 584 DEGs were downregulated.

A heat map depicting the relative expression level of DEGs between the two strains, SD-BZF-19 (OS-1, OS-2, and OS-3) and SD-BZF-19-G14 (VF-1, VF-2, and VF-3), was presented in Figure 4A. Compared with SD-BZF-19, 1244 DEGs were detected in SD-BZF-19-G14, of which 660 (53.05%) were up-regulated and 584 (46.95%) were down-regulated (Figure 4B).

**Figure 5:**
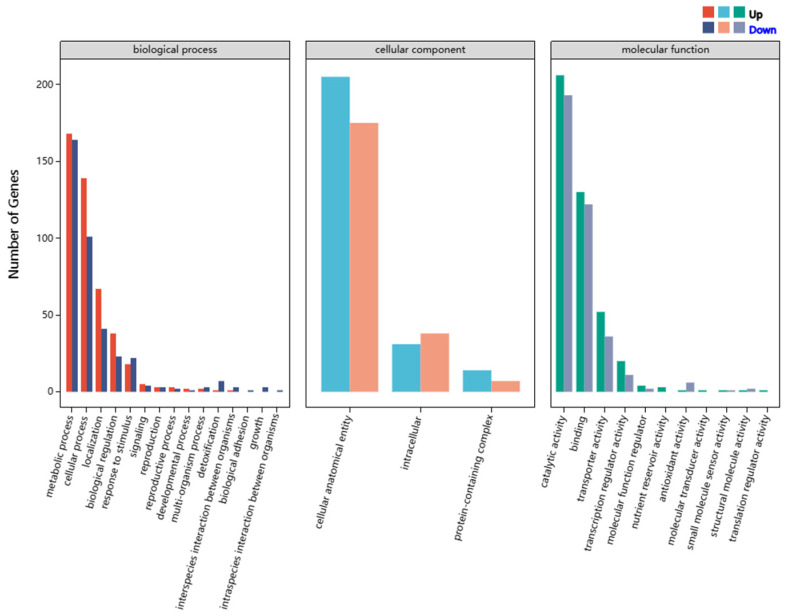
Gene Ontology (GO) classification analysis of DEGs in the two strains, SD-BZF-19 and SD-BZF-19-G14.

DEGs annotated in the GO analysis were classified into three major functional ontologies, namely biological process, cellular component, and molecular function (Figure 5). Among them, 826 DEGs were annotated to biological process, with 447 DEGs being upregulated and 379 DEGs being downregulated; 470 DEGs were annotated to cellular component, with 250 DEGs being upregulated and 220 DEGs being downregulated; 793 DEGs were annotated to molecular function, with 420 DEGs being upregulated and 373 DEGs being downregulated. Amino acid transport (GO: 0006865) (biological process), monooxygenase activity (GO: 0004497) (molecular function), and iron ion binding (GO: 0005506) (molecular function) were the top three GO enrichments according to the *q* value.

**Figure 6:**
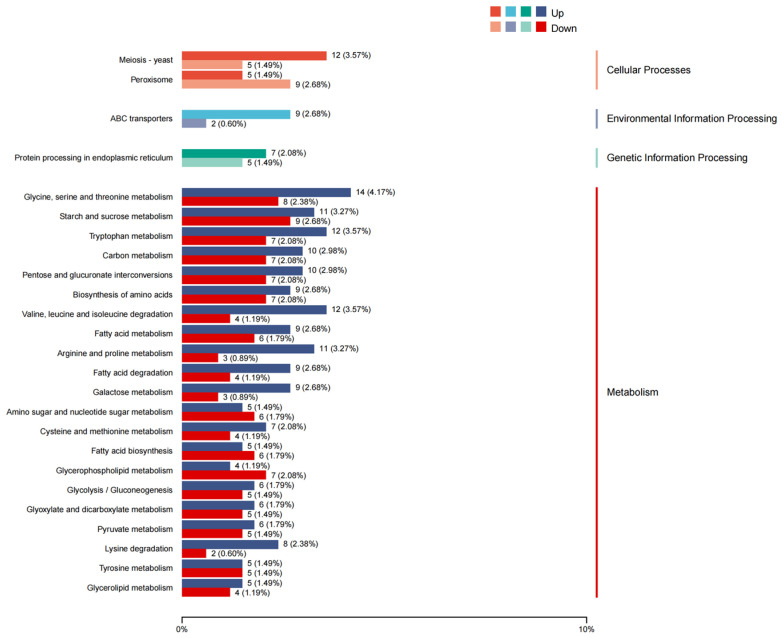
Kyoto Encyclopedia of Genes and Genomes (KEGG) pathways classification analysis of DEGs in the two strains, SD-BZF-19 and SD-BZF-19-G14.

A total of 594 DEGs were annotated to KEGG pathways and classified into four major functional categories, namely cellular processes (46 DEGs), environmental information processing (18 DEGs), genetic information processing (51 DEGs), and metabolism (479 DEGs). According to the *q* value, the three pathways with the most significant difference were pentose and glucuronate interconversions (ko00040), galactose metabolism (ko00052), and starch and sucrose metabolism (ko00564) (Figure 6).

### 3.5. Influence of AaBRV1 on Its Host DEGs Related to MFS Multidrug Transporters and CYP450

Pfam database annotated that the number of DEGs related to MFS and CYP450 was 36 (Table 2) and 28 (Table 3), respectively. The genes mentioned above are related to fungicide resistance. Compared with the AaBRV1-infected strain SD-BZF-19, 16 of 36 DEGs related to MFS were upregulated (1.22-fold to 5.84-fold) and 20 of 36 DEGs related to MFS were downregulated (1.05-fold to 4.01-fold) in the AaBRV1-free strain SD-BZF-19-G14. Relative to the AaBRV1-infected strain SD-BZF-19, ten of 28 DEGs related to CYP450 were upregulated (1.52-fold to 3.18-fold), and 18 of 28 DEGs related to CYP450 were downregulated (1.12-fold to 9.01-fold) in the AaBRV1-free strain SD-BZF-19-G14.

### 3.6. Validation of Differentially Expressed Genes (DEGs)

**Figure 7:**
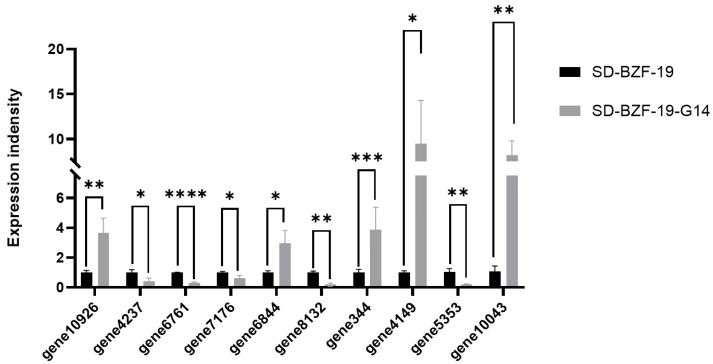
Validation of the transcriptome data obtained for two strains of *A.* *alternata*, SD-BZF-19 and SD-BZF-19-G14, using reverse transcription-quantitative polymerase chain reaction (RT-qPCR). Information on the 10 differentially expressed genes (DEGs) was provided in detail in Table S2. Stars indicate different levels of significant difference between the two strains as determined by the *t*-test using GraphPad Prism version 9.0 software (*, *p* < 0.05; **, *p* < 0.01; ***, *p* < 0.001; ****, *p* < 0.0001).

Ten randomly selected DEGs, including MFS and CYP450-related genes, were subjected to RT-qPCR analysis to validate the expression results obtained in the transcriptome data. Results of the RT-qPCR analysis indicated that among the 10 DEGs in strain SD-BZF-19-G14, the expression of five DEGs was upregulated from 1.52-fold to 2.51-fold, and the remaining five DEGs were downregulated from 1.12-fold to 2.86-fold, relative to their expressions in strain SD-BZF-19 (Figure 7), which was consistent with the transcriptome data.

## 4. Discussion

In recent years, an increasing number of researches focus on the effect of mycoviruses on their fungal hosts and interaction mechanism of mycoviruses with their host fungi [43]. With the development of transcriptome sequencing technologies, the molecular mechanisms of mycoviruses affecting phenotype, virulence, fungicide resistance, and other aspects of their hosts were gradually deepened [17,44,45]. In this study, AaBRV1-free strain SD-BZF-19-G14 were obtained from AaBRV1-infected strain SD-BZF-19 by single hyphal tip culture method combined with high temperature treatment. Compared with strain SD-BZF-19-G14, following AaBRV1 infection, colony color of strain SD-BZF-19 changed, and colony growth rate, dry weight of mycelial biomass, and sensitivity to difenoconazole, fludioxonil, and tebuconazole of strain SD-BZF-19 all decreased. However, the virulence of strain SD-BZF-19 was not significantly different from strain SD-BZF-19-G14. A total of 1244 DEGs were identified in a comparative transcriptome analysis between the two strains, SD-BZF-19 and SD-BZF-19-G14. Notably, Pfam database annotated that the number of DEGs related to MFS and CYP450 was 36 and 28, respectively. These findings expanded our knowledge of the ecology and evolution of viruses in the family *Botybirnaviridae* and provided new insights into the interactions between the mycovirus AaBRV1 and its fungal host *A. alternata* SD-BZF-19.

In related studies, botybirnaviruses were eliminated by single conidium method [8], hyphal tip method [12], or protoplast regeneration method [17]. Additionally, botybirnaviruses could be transmitted to other strains by protoplast fusion [9], protoplast transfection [2,3,4], or pairing culture [8,17]. AaBRV1-free strain SD-BZF-G14 were obtained by single hyphal tip culture method combined with high-temperature treatment, but its horizontal transmission ability remains to be examined in the future. In the future, we could access whether AaBRV1 could transmit to other species of *Alternaria* by protoplast fusion, protoplast transfection, and pairing culture to further explore the effect of AaBRV1 on other species of *Alternaria*.

BpRV1 [2], SsBRV1 [3], SsBRV2 [4], and BmBRV1-BdEW220 [8] reduced colony growth rate of their fungal hosts, AaBbV1 [9], SsBRV3/SZ-150 [11], LbBV1 [12], and DtBRV1 [14] had no significant effect on the colony growth rate of their host fungi, while AaBRV1-AT1 increased the colony growth rate of its host fungus [17]. BpRV1 [2], SsBRV2 [4], and BmBRV1-BdEW220 [8] caused the abnormal colony morphology of their hosts, other known botybirnaviruses had no significant effect on the colony morphology of their host fungi. BpRV1 [2], SsBRV1 [3], SsBRV2 [4], and BmBRV1-BdEW220 [8] could cause hypovirulence on their host fungi, and AaBRV1-AT1 [17] conferred hypervirulence on its host fungus, while AaBbV1 [9], SsBRV3/SZ-150 [11], and DtBRV1 [14] almost did not affect the host virulence. Notably, LbBV1 could reduce virulence of *B. cinerea* strain t-459, but had no effects on virulence of *L. biglobosa* strain GZJS19 [12].

Genes related to MFS transporters and CYP450 have been reported to be associated with pathogen resistance to fungicides. The resistance of *Candida albicans* strains to fluconazole is caused by the upregulation of gene *MDR1* [46]. The overexpression of the CYP450 gene could increase azole resistance of fungi [28]. Mycoviruses could increase/decrease the sensitivity of their hosts to fungicides by disturbing gene expression or metabolism of their host fungi [17,24]. The expression level of one DEG-encoding sterol 14-alpha demethylase and four DEGs encoding siderophore iron transporters were significantly upregulated in AaBRV1-AT1-infected strain *A. tenuissima* TJ-NH-51S-4 [17]. PuRV2 infection might enhance the sensitivity of *G. ultimum* to metalaxyl by decreasing the expression of ABC-type transporter genes [24]. In this study, AaBRV1 infection decreased the sensitivity of SD-BZF-19 to difenoconazole, fludioxonil, and tebuconazole by regulating the expression of MFS transporter-related genes, thereby enhancing drug efflux capacity. At the same time, AaBRV1 infection also influenced expression of CYP450-related genes, which is related to drug metabolism [28].

In summary, our study explored the effect of AaBRV1 infection on the biological characteristics of its host fungus, which provides a new perspective for the interaction between botybirnaviruses and their hosts and helps to understand the evolutionary relationship between mycoviruses and their host fungi.

## Figures and Tables

**Table 1:** Summary of sequencing data and alignment results with the reference genome.

Samples	Clean Reads	≥Q30	Mapped Reads	Mapped Reads Ratio
SD-BZF-19 (OS-1)	42,366,376	96.84%	37,749,516	89.10%
SD-BZF-19 (OS-2)	45,057,340	96.92%	40,344,549	89.54%
SD-BZF-19 (OS-3)	41,652,466	96.79%	37,533,659	90.11%
SD-BZF-19-G14 (VF-1)	40,630,300	97.55%	36,727,212	90.39%
SD-BZF-19-G14 (VF-2)	43,131,488	96.90%	38,787,040	89.93%
SD-BZF-19-G14 (VF-3)	40,859,104	97.39%	36,183,361	88.56%

**Table 2:** Pfam database annotated 36 DEGs to MFS.

#ID	log_2_FC	Regulated	Pfam_Annotation
gene12067	−2.7290	down	Major Facilitator Superfamily
gene3093	−1.8004	down	Major Facilitator Superfamily
gene6558	−2.1461	down	Major Facilitator Superfamily
gene1907	1.7180	up	Major Facilitator Superfamily
gene3128	−1.5539	down	Major Facilitator Superfamily
gene12139	4.7976	up	Major Facilitator Superfamily
gene449	−1.8754	down	Major Facilitator Superfamily
gene13064	2.3708	up	Major Facilitator Superfamily
gene4157	2.1862	up	Major Facilitator Superfamily
gene9346	−2.9509	down	Major Facilitator Superfamily
gene3911	−1.2205	down	Major Facilitator Superfamily
gene8132	−2.2791	down	Major Facilitator Superfamily
gene7671	−4.0058	down	Major Facilitator Superfamily
gene12402	−2.9413	down	Major Facilitator Superfamily
gene7749	−2.1046	down	Major Facilitator Superfamily
gene4382	−1.7318	down	Major Facilitator Superfamily
gene10728	3.4501	up	Major Facilitator Superfamily
gene10144	−1.4685	down	Major Facilitator Superfamily
gene14	−1.2037	down	Major Facilitator Superfamily
gene20	3.2432	up	Major Facilitator Superfamily
gene6734	4.0763	up	Major Facilitator Superfamily
gene4149	2.5069	up	Major Facilitator Superfamily
gene12318	−1.7286	down	Major Facilitator Superfamily
gene2867	5.8406	up	Major Facilitator Superfamily
gene6803	3.5240	up	Major Facilitator Superfamily
gene4338	−1.4488	down	Major Facilitator Superfamily
gene10118	1.3871	up	Major Facilitator Superfamily
gene10927	1.2162	up	Major Facilitator Superfamily
gene11344	2.3732	up	Major Facilitator Superfamily
gene10023	2.3275	up	Major Facilitator Superfamily
gene7724	−1.9912	down	Major Facilitator Superfamily
gene1004	1.6274	up	Major Facilitator Superfamily
gene1015	−1.5243	down	Major Facilitator Superfamily
gene12533	2.8530	up	Major Facilitator Superfamily
gene11267	−1.3916	down	Major Facilitator Superfamily
gene3700	−1.0512	down	Major Facilitator Superfamily

**Table 3:** Pfam database annotated 28 DEGs to CYP450.

#ID	log_2_FC	Regulated	Pfam_Annotation
gene344	1.6649	up	Cytochrome P450
gene12733	−3.5506	down	Cytochrome P450
gene8445	−1.3397	down	Cytochrome P450
gene12537	1.8953	up	Cytochrome P450
gene9662	−5.4342	down	Cytochrome P450
gene10926	1.5227	up	Cytochrome P450
gene2102	−3.7723	down	Cytochrome P450
gene6912	3.1839	up	Cytochrome P450
gene7128	−2.7300	down	Cytochrome P450
gene3654	−4.2753	down	Cytochrome P450
gene2397	−1.9457	down	Cytochrome P450
gene12168	−9.0107	down	Cytochrome P450
gene13312	1.9832	up	Cytochrome P450
gene5352	−5.5725	down	Cytochrome P450
gene9354	−3.9415	down	Cytochrome P450
gene10880	−1.1803	down	Cytochrome P450
gene5571	−4.3013	down	Cytochrome P450
gene7176	−1.1220	down	Cytochrome P450
gene8218	−2.9576	down	Cytochrome P450
gene6073	−3.5630	down	Cytochrome P450
gene10344	−7.0703	down	Cytochrome P450
gene4550	1.5708	up	Cytochrome P450
gene11675	2.0081	up	Cytochrome P450
gene4276	−2.8887	down	Cytochrome P450
gene11233	1.8129	up	Cytochrome P450
gene6652	2.6545	up	Cytochrome P450
gene8442	1.7749	up	Cytochrome P450
gene1667	−1.8225	down	Cytochrome P450

## Data Availability

The transcriptome raw data from the three biological replicates of strains SD-BZF-19 and SD-BZF-19-G14 were deposited in the NCBI Sequence Read Archive (SRA) database under the accession number PRJNA1250114.

## Supplementary Materials

The following supporting information can be downloaded at: https://www.mdpi.com/article/10.3390/jof11050376/s1. Table S1: Detailed information of 14 reported botybirnaviruses; Table S2: Differentially expressed genes (DEGs) information used for reverse transcription-quantitative polymerase chain reaction (RT-qPCR); Table S3: Primers used for reverse transcription-quantitative polymerase chain reaction (RT-qPCR) analysis of ten randomly selected differentially expressed genes (DEGs) identified in the transcriptome data in this study.
