# Supplementary material for: Alternaria alternata botybirnavirus 1 (AaBRV1) Infection Affects the Biological Characteristics of Its Host Fungus Alternaria alternata
Source: J Fungi (Basel). 2025 May 15;11(5):376. doi: 10.3390/jof11050376 (PMC12113547; doi:10.3390/jof11050376)
Supplement: Supplementary file 1 [file jof-11-00376-s001.zip › Table S1.pdf]

Table S1. Detailed information of 14 reported botybirnaviruses.

Virus name	Abbreviation	Host	Year	Novel strain	Reference
Botrytis porri RNA virus 1	BpRV1	*Botrytis porri* strain GarlicBc-72	2012	/	[2]
Sclerotinia sclerotiorum botybirnavirus 1	SsBRV1	*Sclerotinia sclerotiorum* strain SCH941	2015	/	[3]
Sclerotinia sclerotiorum botybirnavirus 2	SsBRV2	*Sclerotinia sclerotiorum* strain AH16	2016	/	[4]
Soybean leaf-associated botybirnavirus 1	SlaBRV1	soybean	2016	/	[5]
Alternaria botybirnavirus 1	ABRV1	*Alternaria* sp. strain SCFS-3	2017	/	[6]
Bipolaris maydis botybirnavirus 1	BmBRV1	*Bipolaris maydis* strain JZ11	2018	BmBRV1-BdEW220	[7-8]
Alternaria alternata botybirnavirus 1	AaBbV1	*Alternaria alternata* strain 4a	2019	/	[9]
Botrytis cinerea botybirnavirus 1	BcBRV1	*Botrytis cinerea* strain CCg427	2019	/	[10]
Sclerotinia sclerotiorum botybirnavirus 3	SsBRV3/SZ-150	*Sclerotinia sclerotiorum* strain SZ-150	2019	/	[11]
Leptosphaeria biglobosa botybirnavirus 1	LbBV1	*Leptosphaeria biglobosa* strain GZJS-19	2022	/	[12]
Hymenoscyphus fraxineus botybirnavirus 1	HfBRV1	*Hymenoscyphus fraxineus*	2022	/	[13]
Didymella theifolia botybirnavirus 1	DtBRV1	*Didymella theifolia* strain CJP4-1	2023	/	[14]
Alternaria botybirnavirus 2	ABRV2	*Alternaria* sp. strain XC21-21C	2024	/	[15]
Alternaria alternata botybirnavirus 1	AaBRV1	*Alternaria alternata* strain SD-BZF-19	2019	AaBRV1-AT1	[16-17]
