# Supplementary material for: Alternaria alternata botybirnavirus 1 (AaBRV1) Infection Affects the Biological Characteristics of Its Host Fungus Alternaria alternata
Source: J Fungi (Basel). 2025 May 15;11(5):376. doi: 10.3390/jof11050376 (PMC12113547; doi:10.3390/jof11050376)
Supplement: Supplementary file 1 [file jof-11-00376-s001.zip › Table S2.pdf]

Table S2. Differentially expressed genes (DEGs) information used for reverse transcription-quantitative polymerase chain reaction (RT-qPCR).

#ID	Pfam_annotation	NR_annotation	regulated	log_2FC	qRT-PCR ($2^{-\Delta\Delta CT}$)	
					SD-BZF-19	SD-BZF-19-G14
gene10926	Cytochrome P450	benzoate 4-monooxygenase cytochrome P450	up	1.5227	1.0094	3.6637
gene4237	ABC transporter	P-loop containing nucleoside triphosphate hydrolase protein	down	-1.8288	1.0120	0.3989
gene6761	Zinc finger, C2H2 type	zinc finger protein OZF	down	-1.9560	1.0013	0.3264
gene7176	Cytochrome P450	cytochrome P450	down	-1.1220	1.0063	0.6183
gene6844	ABC-2 type transporter	ATP-binding cassette transporter-like protein	up	2.1439	1.0051	2.9583
gene8132	Major Facilitator Superfamily	MFS general substrate transporter	down	-2.2791	1.0037	0.1828
gene344	Cytochrome P450	cytochrome P450 monooxygenase-like protein	up	1.6649	1.0177	3.8769
gene4149	Major Facilitator Superfamily	MFS general substrate transporter	up	2.5069	1.0076	9.4999
gene5353	Glycosyl hydrolase family 10	glycoside hydrolase	down	-2.8630	1.0410	0.2157
gene10043	Uncharacterized protein conserved in bacteria	hypothetical protein	up	2.2177	1.0640	8.1806
