# Supplementary material for: Alternaria alternata botybirnavirus 1 (AaBRV1) Infection Affects the Biological Characteristics of Its Host Fungus Alternaria alternata
Source: J Fungi (Basel). 2025 May 15;11(5):376. doi: 10.3390/jof11050376 (PMC12113547; doi:10.3390/jof11050376)
Supplement: Supplementary file 1 [file jof-11-00376-s001.zip › Table S3.pdf]

Table S3. Primers used for reverse transcription-quantitative polymerase chain reaction (RT-qPCR) analysis of ten randomly selected differentially expressed genes (DEGs) identified in the transcriptome data in this study.

Gene name	Primer name	Sequence (5'-3')
gene10926	10926-1F	GTCACATTTGGTCTTGGAGGCA
	10926-1R	TGTTGAGGTCGTGTTTGTTACGC
gene4237	4237-1F	TGCCCACGCTTCTATCCTCC
	4237-1R	GATCAACTGTGATTTCGTCCAACA
gene6761	6761-1F	TTCGCTACTGATGTGGACACTCTT
	6761-1R	GGCTTCTGGGCATTCTTTGAT
gene7176	7176-2F	CAACTGCTCTTTGACCTCGCTAC
	7176-2R	TCGCTTGGATGGCAATAACCT
gene6844	6844-1F	CGCAACGCCTCGCTCAT
	6844-1R	TGCTGGAAGAAGTGGGTGGA
gene8132	8132-2F	GGAGGAAGAAATGCGGATGG
	8132-2R	CGAATGATGGTCGGCAAGAAA
gene344	344-1F	CAGTCGCAAGAGTGGATTAGTGAA
	344-1R	TTGGGATAGTGGGTGGCAGA
gene4149	4149-1F	ACTACGCCTGGAACTCTTACTTTACC
	4149-1R	GCTGATGACGAGACCGACTGAC
gene5353	5353-1F	AAACAGGTCATGGGTAGATACAAGGG
	5353-1R	CGGAAGGCGATGGGAAGGT
gene10043	10043-1F	ATCCCATCGCCGCTATCC
	10043-1R	TGCTCTGCCGCCCACTT
HIS3	H3-1a	ACTAAGCAGACCGCCCGCAGG
	H3-1b	GCGGGCGAGCTGGATGTCCTT
